# The role of ZC3H14 (MSUT2) in neurodevelopment and tauopathies

**DOI:** 10.1093/brain/awag245

**Published:** 2026-07-16

**Authors:** Grace Shelby, Anita H Corbett, Roy R Parker

**Affiliations:** Department of Biochemistry, BioFrontiers Institute, University of Colorado Boulder, Boulder, CO 80303, USA; Department of Biology, Emory University, Atlanta, GA 30322, USA; Department of Biochemistry, BioFrontiers Institute, University of Colorado Boulder, Boulder, CO 80303, USA; Howard Hughes Medical Institute, University of Colorado Boulder, Boulder, CO 80303, USA

**Keywords:** RNA binding protein, ZC3H14, neurodevelopment, tau, RNA, Alzheimer’s disease

## Abstract

The processes of neurodevelopment and neuropathology can be influenced by RNA binding proteins. The RNA binding protein ZC3H14 (also referred to as MSUT2) is of particular interest for two reasons. First, homozygous loss-of-function alleles in *ZC3H14* are associated with an inherited form of intellectual disability. Second, in model systems, loss of ZC3H14 can suppress the toxicity of tau aggregates, which are hallmark diagnostic features of Alzheimer’s disease and other neurodegenerative diseases referred to as ‘tauopathies’.

In this review, we discuss both the normal functions of ZC3H14 and how alterations in these functions may affect cognitive function or tau pathology. Although not fully understood, an emerging theme is that alterations in RNA regulation attributable to ZC3H14 loss can affect neuronal function.

## Teaser

Loss of function of the RNA binding protein ZC3H14 has been associated with intellectual disability and suppression of tau toxicity in model systems. Shelby *et al.* review the functions of ZC3H14 and discuss how altered RNA regulation may contribute to neuronal dysfunction, cognitive impairment, and tau-related neurodegenerative disease.

## Introduction

Humans are subject to a multitude of diseases of the nervous system, including hereditary diseases of neurodevelopment or function, in addition to neurodegenerative diseases in which neurons lose function over time. A key research goal is to understand the molecular defects and alterations that underlie these neurological diseases, in addition to genetic and/or chemical changes that have the potential to prevent or mitigate such neurological dysfunction.

Numerous RNA binding proteins are required for proper neurological function and are associated with neurological disease in at least two broad manners.^1^ In some cases, the loss or gain of function of an RNA binding protein can lead to disease. For example, loss-of-function (LOF) mutations in the survival motor neuron (*SMN*) gene, which assists in small nuclear ribonucleoprotein assembly, lead to spinal muscular atrophy.^2,3^ Likewise, loss of components of the mRNA translation repression and degradation machinery leads to intellectual disability.^4,5^ In these cases, the aberrant neurological development can be understood as defects in ribonucleoprotein (RNP) regulatory mechanisms. In other cases, the aggregation of RNA binding proteins can trigger neurodegenerative diseases. For example, aggregation-prone variants of the FUS, or TDP-43, proteins can lead to frontotemporal lobar degeneration and/or amyotrophic lateral sclerosis.^6-9^ Cytoplasmic aggregation of these RNA binding proteins can reduce their nuclear concentration, leading to aberrant polyadenylation and splicing, thereby promoting neurodegeneration.^10,11^

Tauopathies, which are a class of neurodegenerative diseases characterized by the aggregation of the protein tau, are also associated with the dysfunction of RNA regulation and RNA binding proteins.^12,13^ In tauopathies, several RNA binding proteins mislocalize to tau aggregates and exhibit dysfunction, including but not limited to hnRNPA1B2, TIA1, SRRM2 and ZC3H14.^14-17^ In addition, the formation of tau aggregates can lead to defects in RNA metabolism, including splicing, nonsense-mediated decay and translation. However, the potential contributions of these changes in RNA processing to disease development are unclear.^14,15,18^

The ZC3H14 RNA binding protein is particularly interesting with respect to neurological function for two reasons. First, individuals with homozygous LOF mutations in *ZC3H14* show intellectual disability, demonstrating that this protein plays an important role in neuronal development and/or function.^19^ Second, decreased ZC3H14 function suppresses neuronal pathology by reducing tau aggregation,^20,21^ thereby alleviating neurodegenerative disease in model systems.^13^ In this review, we cover the normal functions of ZC3H14 and discuss how alterations in those processes might lead to intellectual disability and to suppression of tau pathology.

## Properties of ZC3H14

ZC3H14, also referred to as mammalian suppressor of tauopathy (MSUT2), is a conserved zinc finger RNA binding protein that binds A-rich stretches of RNA, presumably including the poly(A) tails of mRNAs, with high affinity.^22^ ZC3H14 is functionally related to the *Saccharomyces cerevisiae* orthologue Nab2 and has orthologues in other eukaryotes, including *Drosophila* (Nab2) and *Caenorhabditis elegans* (SUT-2).^19,20,22^ ZC3H14 binds to poly(A) RNA sequences through Cys_3_His tandem zinc finger domains (Fig. 1A).^22^ ZC3H14 also contains a nuclear localization signal and is predominantly localized to the nucleus, specifically in nuclear speckles.^24^ The proline–tryptophan–isoleucine-like domain in ZC3H14 is likely to mediate interactions with components of the nuclear pore complex, as has been demonstrated for the budding yeast orthologue, Nab2.^25,26^ Recent analysis suggests a potential role for the glutamine-rich domain in stress-dependent condensate formation, which can regulate RNA export from the nucleus, at least in budding yeast.^27^

**Figure 1:**
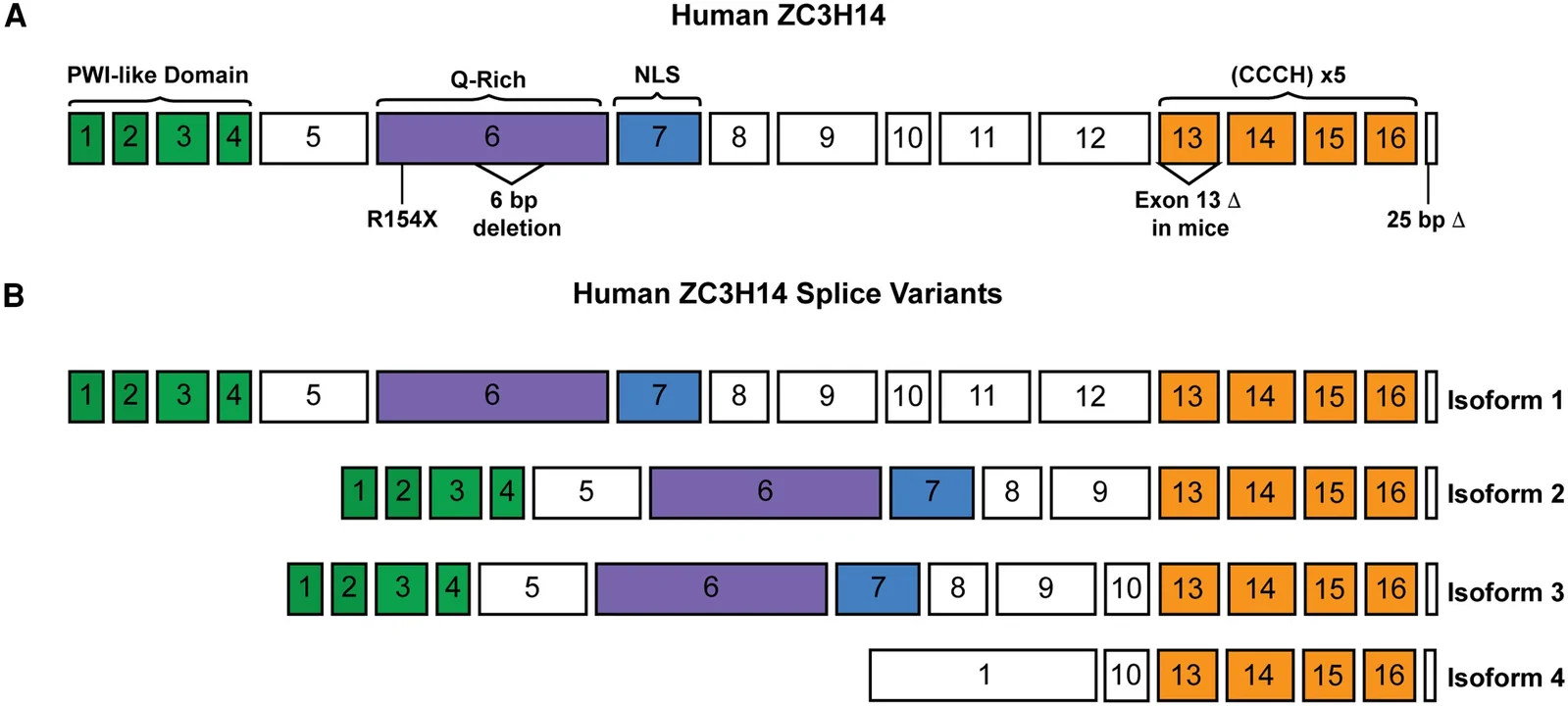
***ZC3H14* domains and splice variants.** (**A**) This diagram depicts the total exons within the human *ZC3H14* gene along with the following annotated domains: An N-terminal proline–tryptophan–isoleucine (PWI)-like fold (green), a glutamine (Q)-rich domain (purple), a predicted classical nuclear localization signal (blue) and a C-terminal Cys_3_His (CCCH) tandem zinc finger domain (orange). The Arg154Stop (R154X) mutation in exon 6 and a separate 25 bp deletion 16 bp downstream from the 3′ end of exon 16 linked to intellectual disability are indicated.^19^ To illustrate the mouse models that have been generated, a 6 bp deletion in exon 6 and an exon 13 deletion are depicted.^21,23^ (**B**) These diagrams represent the different splice variants of the human *ZC3H14* gene, which encode ZC3H14 isoforms 1–4.

In mammalian cells, four isoforms of ZC3H14 are expressed: three long isoforms (isoforms 1–3) and one short isoform (isoform 4) (Fig. 1B).^24^ Isoforms 1–3 are expressed ubiquitously and are localized to the nucleus at steady state, whereas isoform 4 is localized in the cytoplasm and is expressed mainly in the testes and neurons. Interestingly, as illustrated in Fig. 1A, the best-characterized mutations that give rise to intellectual disability in patients lead to the loss of isoforms 1–3, but are predicted to leave the cytoplasmic isoform 4 intact.^19^ This implies that isoforms 1–3 are important for neuronal development and/or function.

ZC3H14 appears particularly significant in neurons. This idea is supported by the high expression of ZC3H14 in neurons compared with most other cell types.^24^ In addition, although the *Drosophila* orthologue, referred to as Nab2, is essential for viability, neuron-specific expression is sufficient to restore viability.^19^ Importantly, human ZC3H14 isoform 1 can also rescue viability of *Nab2* null flies when expressed specifically in *Drosophila* neurons, demonstrating the functional conservation of ZC3H14 and *Drosophila* Nab2.^28^ Additionally, Nab2 is essential for viability of budding yeast.^29^ In contrast, ZC3H14 is not essential for viability in mice or *C. elegans*.^20,28^ These lines of evidence argue that ZC3H14 has a critical role within neurons.

## Functions of ZC3H14

ZC3H14 can have multiple effects on RNA biogenesis. As detailed later, the multiple functions attributed to ZC3H14 may all be consistent with ZC3H14 binding to internal A-rich regions of RNAs or poly(A) tails (Fig. 2A).

**Figure 2:**
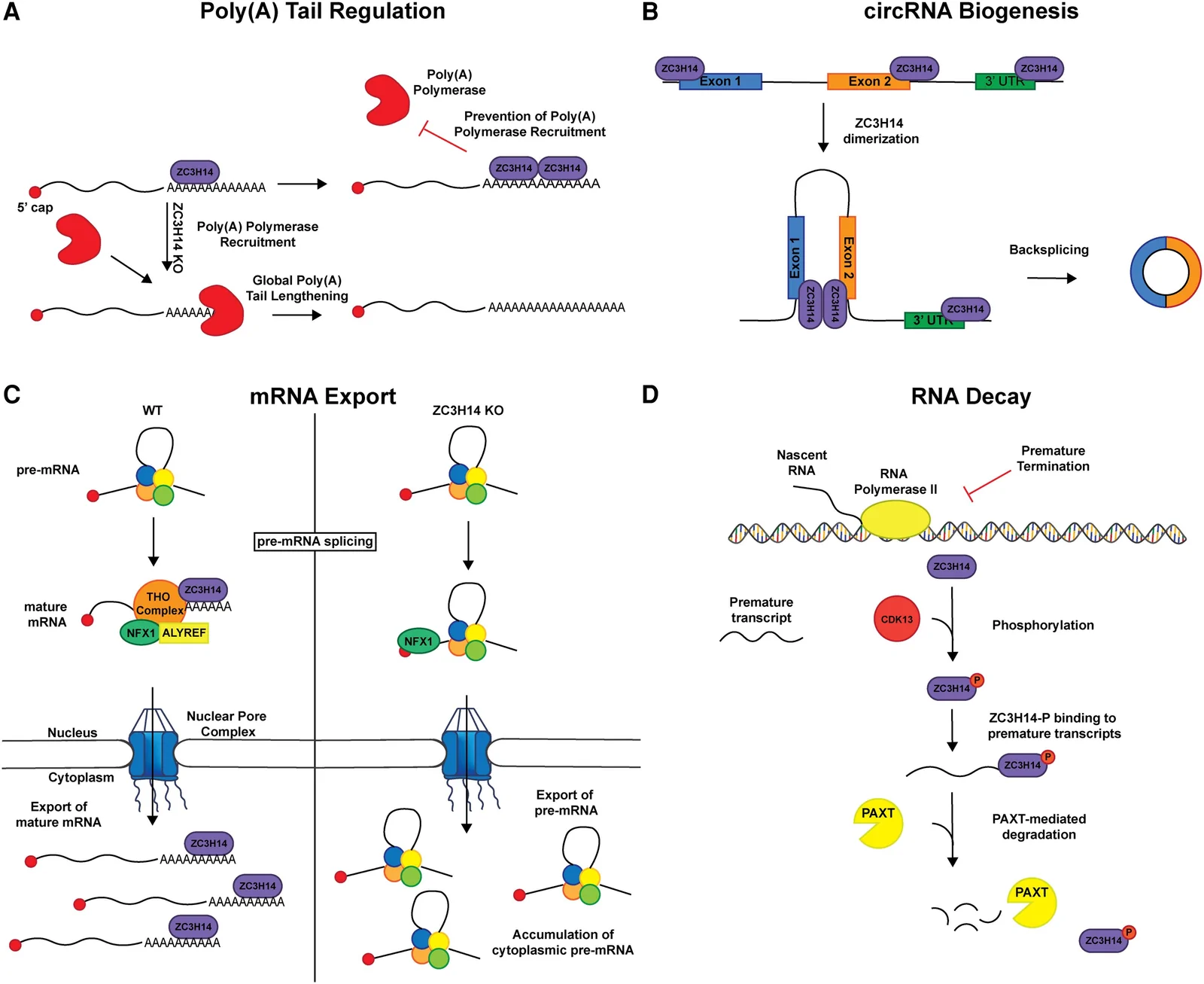
**ZC3H14 regulates RNA biogenesis through regulation of mRNA poly(A) tail length, circRNA biogenesis, mRNA export and RNA degradation**. (**A**) ZC3H14 is proposed to bind to and regulate the length of mRNA poly(A) tails through dimerization that can decrease recruitment of poly(A) polymerases.^30^ Additionally, loss of ZC3H14 leads to global poly(A) tail lengthening.^22^ (**B**) ZC3H14 promotes circRNA biogenesis by binding to exon–intron boundaries, dimerizing and, subsequently, promoting RNA circularization.^23^ The binding of ZC3H14 to the 3′ untranslated region (UTR) stabilizes this interaction.^23^ (**C**) ZC3H14 is proposed to regulate mRNA export by interacting with the THO complex and associated factors, thereby promoting the interaction of mature mRNA with NFX1 and other export factors.^31^ Loss of ZC3H14 leads to premature interaction of pre-mRNA with export factors and, consequently, accumulation of pre-mRNAs in the cytoplasm.^31^ (**D**) CDK13 phosphorylates ZC3H14 when RNA polymerase II terminates transcription prematurely.^32^ This phosphorylation event promotes ZC3H14-mediated recruitment of the PAXT complex to degrade the prematurely terminated transcript.^32^ KO = knockout.

One conserved function of ZC3H14 is to regulate the poly(A) tail length of RNAs. This role was first suggested by defects in the budding yeast Nab2 orthologue, leading to hyperadenylation of RNA.^33^ Moreover, knockdown (KD) of ZC3H14 in mouse neuronal N2A cells leads to increased overall poly(A) tail length.^31,34^ More specifically, a mouse model with deletion of exon 13, the first common exon present in all ZC3H14 isoforms, shows extended poly(A) tails in RNA prepared from the hippocampus.^28^ Deletion of exon 13 abolishes ZC3H14 protein levels.^21^ Likewise, loss of Nab2 in *Drosophila* leads to increased poly(A) tail length.^19^ Experiments with budding yeast Nab2 suggest that this protein limits poly(A) length by binding nascent poly(A) tails, multimerizing and thereby limiting the recruitment of the polyadenylation machinery.^35^ Thus, control of poly(A) tail length seems to be one conserved function of Nab2/ZC3H14. This conserved function is important because extended poly(A) tails on RNA can prevent export from the nucleus, confer stability in the cytosol and/or increase translational output depending on the context.

In mammals, ZC3H14 can also directly affect pre-mRNA splicing by binding exon–intron boundaries or 3′ untranslated region sequences, dimerizing, then promoting backsplicing to generate circRNAs (Fig. 2B).^23^ This role for ZC3H14 is supported by observations that ZC3H14 can interact with splicing machinery, and yeast or mice lacking ZC3H14 show decreased levels of circRNAs.^23^ Additionally, recent work demonstrates that ZC3H14/SUT-2 is involved in SOS splicing in *C. elegans*, where DNA transposable elements are spliced from mRNAs.^36^ These observations raise the possibility that alterations in ZC3H14 can impact pre-mRNA processing in unexpected ways.

ZC3H14 can also affect export of mRNAs from the nucleus (Fig. 2C). This role was first supported by observations that yeast cells defective for Nab2 function accumulate poly(A) RNA within the nucleus.^33^ However, more recent work implicates ZC3H14 in quality control of RNA export from the nucleus. Studies of mouse brain lysate revealed that ZC3H14 interacts with the THO complex, which is required for mRNA export.^31^ KD of THO components, in addition to ZC3H14, leads to extended bulk poly(A) tails. Strikingly, when either THO1 or ZC3H14 is knocked down, mRNAs that interact with both ZC3H14 and the THO complex accumulate in the cytosol as pre-mRNAs.^31^ This finding shows that ZC3H14 might limit the export of incompletely processed mRNAs in some manner.^31^ One possibility is that ZC3H14 could regulate the interaction between RNA and the RNA export factor NFX1, which binds mature mRNA and facilitates its export to the cytoplasm.^37^ Loss of THOC1 and ZC3H14 leads to increased interaction between NFX1 and both *Atp5g1* and *Psd95* pre-mRNA.^31^ This finding suggests that the THO complex and ZC3H14 work together and independently to regulate the proper export of mature mRNA, and the loss of either protein may lead to the premature export of pre-mRNAs.

ZC3H14 also affects RNA degradation within the nucleus (Fig. 2D). For example, in zebrafish, CDK13-mediated phosphorylation of ZC3H14 promotes the degradation of prematurely terminated mRNAs, such as those from the *CPA* gene.^32^ In this case, ZC3H14 interacts with the PAXT complex, which recruits the nuclear RNA exosome for RNA degradation. ZC3H14 may also recognize and promote the degradation of unspliced pre-mRNAs. In budding yeast, rapid depletion of Nab2 from the nucleus leads to a global loss of cellular mRNAs.^38^ In contrast to depletion of Nab2, conditional Nab2 mutant cells accumulate unspliced pre-mRNA, and Nab2 can interact with some splicing factors.^39^ This observation suggests a model whereby Nab2 binding to unspliced pre-mRNAs retains those RNAs in the nucleus and eventually recruits the nuclear RNA exosome to mediate their degradation. Likewise, in mammalian cells, nuclear ZC3H14 preferentially binds the pre-mRNA of *ATP5G1* and retains that species in the nucleus.^40^

Taken together, these studies reveal that ZC3H14 is a multifunctional protein that binds to nuclear pre-mRNAs through poly(A)-rich sequences, which may be located at the poly(A) tail or within internal sequences. Upon binding pre-mRNAs, ZC3H14 can influence pre-mRNA splicing, polyadenylation, nuclear degradation and nuclear retention. These studies highlight how this protein can have a variety of effects on the transcriptome through effects on RNA biogenesis.

## ZC3H14 defects are linked to intellectual disability

Humans with homozygous LOF alleles in *ZC3H14* present with an inherited form of autosomal recessive intellectual disability. This link was first identified in a large-scale screen, in which a family with affected children was found to harbour a homozygous nonsense mutation (R154X) in exon 6 of the *ZC3H14* gene (Fig. 1A). Introduction of this premature stop codon produces a truncated ZC3H14 protein that lacks the critical zinc finger, RNA binding domain. In addition, in a second family, a 25 bp deletion 16 bases 3′ of the 3′ end of exon 16 co-segregates with the intellectual disability (Fig. 1A).^19^

The viability of humans with homozygous LOF mutations in *ZC3H14* implies that this gene is not essential in mammals. Consistent with this interpretation, knockout (KO) mice lacking exon 13 (*Zc3h14*^Δ13/Δ13^) or carrying a deletion in exon 6 are viable.^21,23^ Although *Zc3h14* is not essential for mouse viability, *Zc3h14*^Δ13/Δ13^ mice exhibit physical and behavioural abnormalities.^28^ First, *Zc3h14*^Δ13/Δ13^ mice have enlarged lateral ventricles within the brain.^28^ Second, *Zc3h14*^Δ13/Δ13^ mice have impaired learning, as indicated in a water radial arm maze test.^28^ Third, RNA isolated from the hippocampus of *Zc3h14*^Δ13/Δ13^ mice has lengthened poly(A) tails.^28^ Fourth, there are changes in the steady-state levels of some synaptic proteins, which might be attributable to interactions between ZC3H14 and the corresponding mRNA, because RNA immunoprecipitation revealed decreased levels of the corresponding RNAs.^28^ These findings support the conclusion that ZC3H14 is not necessary for viability but is required for proper brain function in mice.^28^ This crucial, but non-essential, role of ZC3H14 in the mammalian brain might explain why humans lacking ZC3H14 are viable but develop intellectual disability.

How the loss of ZC3H14 causes intellectual disability remains unknown. The simplest possibility is that changes in the transcriptome of neurons lead to altered regulation and neuronal dysfunction. However, RNA sequencing of total brains from *Zc3h14* KO mice demonstrates that only a small number of transcripts are affected by loss of ZC3H14.^21,40^ Single-cell metabolically labelled new RNA tagging sequencing (scNT-seq) in mouse primary neurons revealed that ZC3H14 differentially affects gene expression in a cell-type-dependent manner.^41^ In this analysis, levels of transcripts that encode proteins that play roles in protecting against misfolded proteins are increased, and multiple transcripts encoding proteins implicated in tau pathology are decreased.^41^ This observation suggests that ZC3H14 impacts a specific subset of biologically important transcripts that are crucial for proper brain function; however, more work is needed to determine how KD/KO of ZC3H14 leads to changes in gene expression for specific transcripts.

## ZC3H14 loss of function suppresses tau pathology

ZC3H14 LOF mutations were first suggested to suppress tau pathology when the loss of SUT-2, the *C. elegans* orthologue of ZC3H14, was identified as a suppressor of the abnormal movement observed in transgenic *C. elegans* expressing human tau.^20,42^ This study demonstrated that loss of SUT-2 reduces tau aggregation *per se*, because *SUT-2* mutants showed reduced accumulation of detergent-insoluble tau.^20^ Loss of SUT-2 in tau transgenic worms also rescued neuronal loss and reduced lifespan owing to the tau transgene.^20,43^ Interestingly, overexpression of SUT-2 in tau transgenic *C. elegans* results in increased insoluble tau accumulation.^16^ Overall, these findings show that SUT-2 increases the susceptibility of *C. elegans* to tau toxicity and that loss of SUT-2 function can reduce tau pathology.

Five observations suggest that the mammalian orthologue of ZC3H14 affects the development of tau pathology in a similar manner. First, loss of ZC3H14 reduces the amount of detergent-insoluble tau in HEK293 cells that express the tau isoform 1N4R.^16^ Additionally, ZC3H14 KD in HEK293 cells expressing the microtubule binding region of tau (K18) show decreased tau aggregation.^44^ Second, knockout of *Zc3h14* in mice expressing the pathogenic P301S tau transgene (PS19 mice) results in reduced neuroinflammation markers, such as GFAP, a marker for reactive astrocytes.^21^ Third, injection of tau isolated from post-mortem Alzheimer’s disease brains into PS19 mice lacking ZC3H14 shows decreased insoluble tau species compared with control mice.^41^ Additionally, PS19 mice lacking ZC3H14 perform better in the Barnes maze test, a hippocampus-dependent test of memory, compared with PS19 control mice. In these experiments, PS19 mice lacking ZC3H14 required fewer trials and located the escape hole in less time. These results indicate that ZC3H14 KO suppresses some of the behavioural defects seen with expression of a pathogenic human tau transgene.^21^ Finally, overexpression of ZC3H14 in PS19 mice increased phosphorylated and detergent-insoluble tau, decreased the number of hippocampal neurons and increased IBA1 staining, a marker for active microglia.^21^ These findings imply that ZC3H14 can affect the development of tau pathology in mammals, with reduced ZC3H14 levels decreasing tau pathogenesis, whereas an increase in ZC3H14 levels leads to increased tau pathology.

A challenge is to understand how the different orthologues of ZC3H14 suppress tau pathology in *C. elegans* and mammalian cells. One possibility is that both *C. elegans* and mammalian ZC3H14 directly bind tau protein and modulate folding, thereby affecting aggregation. Alternatively, both orthologues have similar effects on poly(A) tail length and splicing, which could then have a common shared impact on tau aggregation.

## Does ZC3H14 affect disease progression in humans?

A key unanswered question is whether alterations in the human ZC3H14 protein affect tauopathy development in humans. For example, given that ZC3H14 dosage appears to be correlated with tau progression, one might expect that humans with one LOF allele of *ZC3H14* might show reduced rates of tauopathies, although, to our knowledge, no data exist to address this point. However, immunoblot analysis of brain tissue from the temporal cortex shows lower levels of ZC3H14 in Alzheimer’s disease brain tissue.^16^ Immunostaining of Alzheimer’s disease and control temporal cortex brain tissue also showed that Alzheimer’s disease brain tissue contains less ZC3H14 than control brain tissue.^16^ One interpretation of this observation is that cells with low levels of ZC3H14 are less likely to die in tauopathies and thus there is a reduced level in post-mortem tissue owing to preferential survival of neurons with low ZC3H14.^16^

Another possibility is that ZC3H14 levels are reduced in the Alzheimer’s disease brain owing to co-depletion of ZC3H14 and PABPN1, which is a nuclear poly(A) binding protein that has antagonistic roles to ZC3H14 in both poly(A) tail regulation and RNA decay.^34,45,46^ In Alzheimer’s disease brain tissue, there is decreased immunoreactivity for ZC3H14 and PABPN1 antibodies.^21^ This could be explained by reduced levels of PABPN1 leading to reduced levels of ZC3H14.^21^

## How does ZC3H14 affect tau pathology?

Several models have been proposed for how ZC3H14 might affect tau pathology; however, no clear mechanism has yet been defined. Based on analyses of ZC3H14 LOF conditions, two mechanisms have been proposed for how ZC3H14 could affect tau pathogenesis. First, the effects of ZC3H14 on RNA biogenesis, possibly through regulation of mRNA poly(A) tail length, could lead to increased tau pathology. Second, ZC3H14 might alter the expression of genes that are involved in tau pathogenesis. One challenge across these different models is to explain how increased ZC3H14 levels can also increase tau pathology.

### Poly(A) tails and tau pathology

One possible model is that increases in overall or specific poly(A) tail lengths attributable to the loss of ZC3H14 might alter the formation and/or toxicity of tau aggregates. This possibility is suggested by observations that KD of other proteins that regulate poly(A) tail length can alter tau toxicity. For example, KD of PABPN1, which leads to shorter poly(A) tails,^34,39^ is thought to exacerbate tau pathology, as seen by increased immunoreactivity for phosphorylated tau in HEK293 cells overexpressing human tau.^21^ Alternatively, KD of the CCR4 and PANL-2 deadenylases leads to longer cytoplasmic poly(A) tail length and increased motor deficits in *C. elegans*, associated with tau pathology.^21,47^ In contrast, LOF mutations in *parn-2*, a major nuclear deadenylase in *C. elegans*, suppress tau pathology as assessed by decreased motor deficits.^47^ Taken together, these results suggest that changes in poly(A) lengths can alter the development of tau pathology, but whether this effect is directly attributable to poly(A) tails or to the alterations in the transcriptome caused by these perturbations is unclear. One experiment to assess whether poly(A) RNA alone is sufficient to alter tau pathology was to overexpress a 45-nucleotide-long poly(A) RNA exogenously in transgenic *C. elegans* expressing wild-type 1N4R tau.^48^ Exogenous expression of a poly(A) sequence led to increased tau pathology, consistent with a possible direct effect of poly(A) sequences on tau pathology.^48^

Interestingly, there are contrasting results depending on the subcellular location of the altered poly(A) tail length and the effect of this change on tau pathology (Table 1). An emerging theme is that lengthening of poly(A) tails in the nucleus is correlated with suppression of tau pathology, whereas lengthening of poly(A) tails in the cytoplasm is correlated with exacerbation of tau pathology. Whether lengthened poly(A) tails in the nucleus lead to suppression of tau pathology and, if so, by what mechanism, remains unclear.

**Table 1:** Genetic perturbation of deadenylases and ZC3H14 and the resulting effect on tau aggregation

Genetic change	Organism	Poly(A) length	Tau aggregation	Location
*Zc3h14*Δ	Mouse	Increased	Decreased	Nucleus
*sut-2*Δ	*Caenorhabditis elegans*	Increased	Decreased	Nucleus
*parn-2* LOF	*Caenorhabditis elegans*	Increased	Decreased	Nucleus
*PABPN1* knockdown	HEK293	Decreased	Increased	Nucleus
*ccr4* loss of function	*Caenorhabditis elegans*	Increased	Increased	Cytoplasm
*panl-2* loss of function	*Caenorhabditis elegans*	Increased	Increased	Cytoplasm
Poly(A)_45_	*Caenorhabditis elegans*	45	Increased	Unknown

### ZC3H14 in tau aggregates

ZC3H14 co-localizes with tau aggregates in cell line models of disease in two ways. Specifically, in tau biosensor cells, ZC3H14 accumulates at low levels in cytoplasmic tau aggregates.^17^ In addition, when tau aggregates form in the nucleus, they assemble within nuclear speckles, which are RNA and protein condensates involved in pre-mRNA splicing and export.^17^ When tau aggregates form within nuclear speckles, the organization of the speckle is disrupted, with ZC3H14 localizing to the periphery of the nuclear speckle containing tau aggregates.^17^ There is a possibility that tau, either in cytoplasmic aggregates or in nuclear speckles, might therefore come into contact with poly(A) RNA, for which tau has a higher binding affinity than microtubules (Fig. 3A).^48^ This possible interaction might lead to increased tau aggregation by increasing the concentration of tau unbound to microtubules and/or RNA-bound tau, leading to greater aggregation.

**Figure 3:**
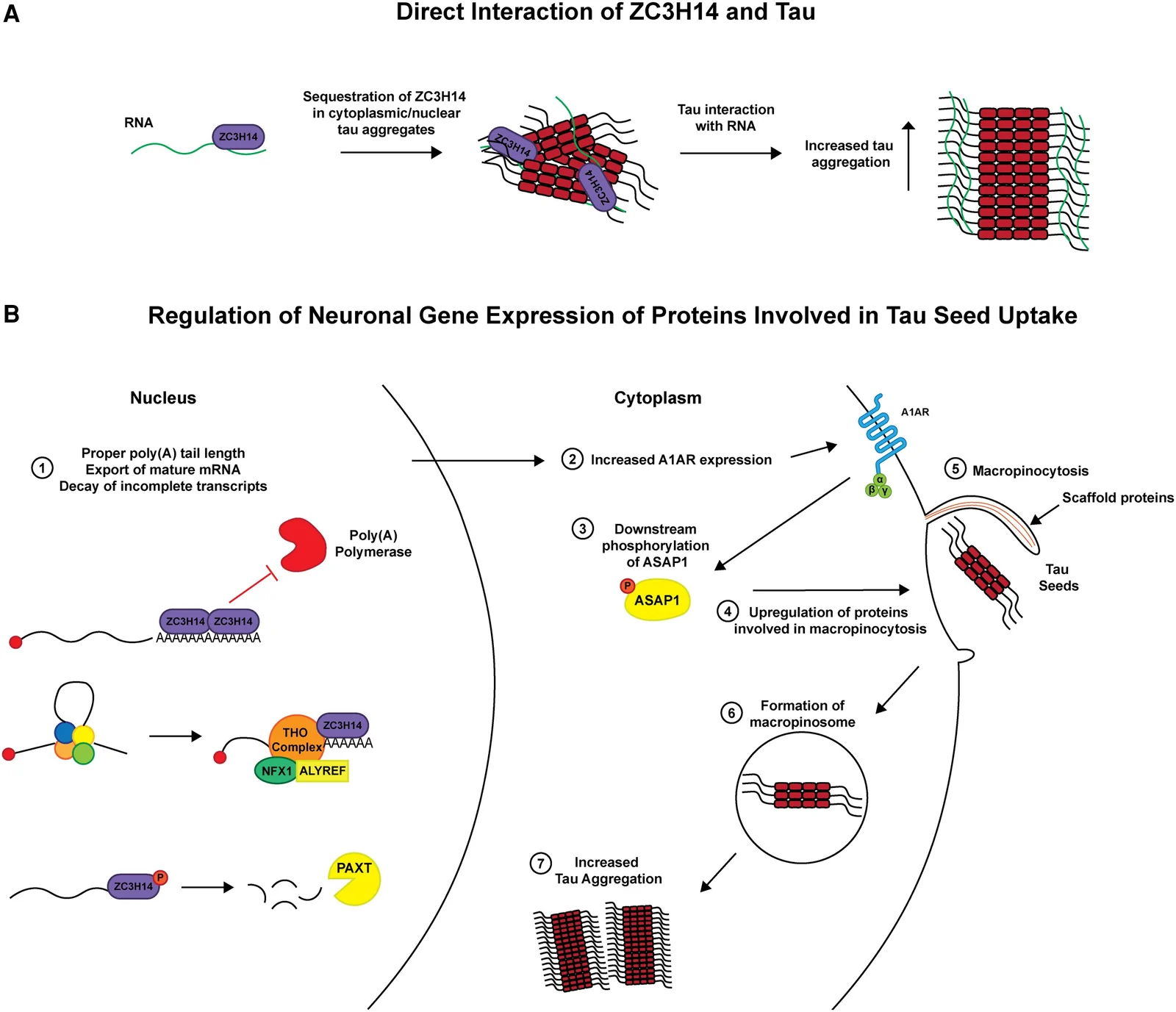
**Proposed mechanisms for how ZC3H14 exacerbates tau pathology.** (**A**) ZC3H14 binds to RNAs and is found in cytoplasmic and nuclear tau aggregates.^17^ The introduction of RNA to the tau protein may induce further fibrillization, because RNA is a known cofactor for tau fibrillization.^49^ (**B**) ZC3H14 also regulates a number of transcripts that encode proteins involved in tau internalization.^41^ Through the multiple roles that ZC3H14 plays in regulating RNA biogenesis, adenosine receptor 1 (A1AR) levels are increased, and downstream phosphorylation of ASAP1 upregulates scaffold proteins involved in macropinocytosis.^41^ Consequently, tau seeds can be internalized via macropinocytosis, thereby increasing tau aggregation within neurons.^41.^

The aggregation of tau in nuclear speckles inhibits mRNA export from the nucleus,^50^ which might contribute to the pathological consequences of tau aggregation. An intriguing possibility is that loss of ZC3H14 might restore mRNA export in this case and could be part of the mechanism by which ZC3H14 loss suppresses tau toxicity.

### ZC3H14 loss results in differential expression of genes implicated in tau pathology

The diverse effects of ZC3H14 on mRNA biogenesis suggest that loss of ZC3H14 could alter the transcriptome and/or proteome, leading to decreased tau pathology. Indeed, loss of ZC3H14 causes changes in the transcriptome of primary mouse neurons with some cell-type-dependent effects.^41^ In the *Zc3h14* KO mouse, multiple transcripts that encode proteins that control endocytosis were altered in parallel with an increase in the level of transcripts encoding proteins that protect against misfolded proteins or oxidative stress.^41^ Other genes, such as *Adora1*, *Apoe* and *Jun*, which are increased at the mRNA level, are implicated in tau pathology.^51-56^ One interpretation of these results is that ZC3H14 regulates the levels of multiple transcripts affecting tau pathology.^41^ Most upregulated genes showed an increased synthesis rate.^41^ Additionally, there is a change in the degradation rate of both increased and decreased transcripts, indicating that ZC3H14-mediated effects on gene expression can alter mRNA stability.^41^ These data suggest that ZC3H14 might modulate tau pathology by altering the expression of genes and/or pathways implicated in tau pathogenesis, including genes that are neuroprotective and harmful.

### ZC3H14 regulates the expression of genes that regulate tau seed internalization

Further analysis of scNT-seq results showed that many transcripts encoding proteins involved in endocytosis, including macropinocytosis, show decreased levels upon loss of ZC3H14.^41^ Two findings support a model in which these factors are involved in tau pathogenesis. First, when anti-sense oligonucleotides were used to KD ZC3H14 in mouse primary neurons, with addition of tau seeds from Ad, corticobasal degeneration or progressive supranuclear palsy brains, the neurons with a decrease in ZC3H14 showed reduced tau pathology.^41^ To determine whether this effect is specific to tau, alpha-synuclein preformed fibrils were used, and no difference in internalization was observed.^41^ Second, internalization of seeds was visualized by using tau seeds that contain a pH-insensitive fluorescent label.^41^ This internalization assay showed that ZC3H14 KD neurons were less efficient at internalizing tau seeds than control neurons.^41^ ZC3H14 KD neurons were also less effective at sequestering tau seeds into acidic compartments when compared with control neurons; however, this could be a result of less tau internalized in cells and not a major function of ZC3H14.^41^ Taken together, these results suggest that loss of ZC3H14 leads to cellular changes that reduce endocytosis, which might limit the spread of tau pathology.

### ZC3H14 loss could regulate tau seed endocytosis via downregulation of adenosinergic receptor expression and downstream ASAP1 signalling

Genes involved in endocytosis showed decreased expression in ZC3H14 KO neurons compared with control neurons. Most interestingly, *Adora1*, which encodes adenosine receptor 1 (A1AR), has a potential link with tauopathy and is involved with neuronal endocytosis (Fig. 3B).^41,53-56^ Reduced expression of A1AR was confirmed by immunoblot.^41^ To investigate the role of A1AR in tauopathy, A1AR was knocked down through the use of anti-sense oligonucleotides in mouse primary neurons, and as a result, there was decreased tau seed internalization.^41^ To investigate further how A1AR might regulate tau pathology, an adenosine agonist and an antagonist, adenosine and caffeine, respectively, were used, and tau seeds were introduced.^41^ Treatment with adenosine leads to increased tau aggregation in mouse primary neurons, whereas caffeine treatment leads to decreased tau aggregation.^41^ In *Zc3h14* KO primary mouse neurons, treatment with caffeine was less effective, with less reduction in tau aggregation compared with wild-type primary mouse neurons, providing further evidence that A1AR is involved in internalizing tau seeds.^41^ Furthermore, KD of A1AR with anti-sense oligonucleotides decreased tau seed internalization in mouse primary neurons, which is similar to the phenotype observed in ZC3H14 KO primary mouse neurons.^41^ These data support the conclusion that A1AR is a downstream gene product regulated by ZC3H14.^41^

## Perspectives and future directions

ZC3H14 is a multifunctional protein that affects many aspects of pre-mRNA biogenesis and function. Given this complex function, future experiments to understand the complexes that ZC3H14 forms and how those complexes might modulate splicing, polyadenylation and mRNA export are crucial. Are there multiple complexes containing ZC3H14 or is there a central assembly that affects many processes?

In addition, loss of ZC3H14 is associated with neurodevelopmental defects. However, the specific molecular events that lead to intellectual disability remain to be identified. Future experiments examining how neurons develop in the absence of ZC3H14 will be important. Does ZC3H14 affect the developmental path of neurons or simply their function once they are fully mature? Are there differences in how ZC3H14 affects different neuronal subtypes, and are certain subtypes more sensitive to the loss of ZC3H14?

Furthermore, understanding how LOF of ZC3H14 rescues tau pathology has important implications. The current data are most consistent with ZC3H14 directly or indirectly promoting the formation of tau aggregates. However, how ZC3H14 loss reduces the amount of tau aggregation is not clear. Although there are several models, none of the current models provides an explanation for how overexpression of ZC3H14 leads to increased tau pathology. Understanding this issue might reveal new insights into how tau pathology develops and provide avenues for possible interventions.

ZC3H14 is also an intriguing therapeutic target for treating tauopathies. In this light, small molecule inhibitors targeting the interaction between ZC3H14 and poly(A) RNA have been identified.^57^ Multiple compounds were identified that bound tightly and selectively to ZC3H14.^57^ However, what effect these compounds might have *in vivo* is not yet known. Developing possible small molecule inhibitors for ZC3H14 is challenging, because ZC3H14 lacks an active site and interactions show evidence of interplay with PABPN1, an essential protein.^58^ Even with these challenges, ZC3H14 is a promising therapeutic target owing to its role in exacerbating tau pathology and the fact that loss of ZC3H14 is not lethal.
